# Real-World Diagnostic Accuracy and Use of Immunohistochemical Markers in Lung Cancer Diagnostics

**DOI:** 10.3390/biom11111721

**Published:** 2021-11-18

**Authors:** Kajsa Ericson Lindquist, Inga Gudinaviciene, Nektaria Mylona, Rodrigo Urdar, Maria Lianou, Eva Darai-Ramqvist, Felix Haglund, Mátyás Béndek, Erika Bardoczi, Katalin Dobra, Hans Brunnström

**Affiliations:** 1 Department of Genetics and Pathology, Laboratory Medicine Region Skåne, SE-221 85 Lund, Sweden; Kajsa.EricsonLindquist@skane.se (K.E.L.); Inga.Gudinaviciene@skane.se (I.G.); nektaria.mylona@skane.se (N.M.); Rodrigo.MunozMitev@skane.se (R.U.); Maria.Lianou@skane.se (M.L.); 2 Division of Pathology, Department of Clinical Sciences, Lund University, SE-221 00 Lund, Sweden; 3 Department of Clinical Pathology and Cytology, Karolinska University Hospital Solna, SE-171 76 Stockholm, Sweden; Eva.Darai-Ramqvist@sll.se (E.D.-R.); felix.haglund@ki.se (F.H.); 4 Department of Oncology-Pathology, Karolinska Institute, SE-171 77 Stockholm, Sweden; 5 Department of Clinical Pathology and Cytology, Karolinska University Hospital Huddinge, SE-141 86 Stockholm, Sweden; matyas.bendek@sll.se (M.B.); erika.bardoczi@sll.se (E.B.); katalin.dobra@ki.se (K.D.); 6 Division of Laboratory Medicine, Department of Pathology, Karolinska Institute, SE-141 86 Stockholm, Sweden

**Keywords:** biopsy, bronchoscopy, cytology, sampling, transthoracic, TTF-1

## Abstract

Objectives: Accurate and reliable diagnostics are crucial as histopathological type influences selection of treatment in lung cancer. The aim of this study was to evaluate real-world accuracy and use of immunohistochemical (IHC) staining in lung cancer diagnostics. Materials and Methods: The diagnosis and used IHC stains for small specimens with lung cancer on follow-up resection were retrospectively investigated for a 15-month period at two major sites in Sweden. Additionally, 10 pathologists individually suggested diagnostic IHC staining for 15 scanned bronchial and lung biopsies and cytological specimens. Results: In 16 (4.7%) of 338 lung cancer cases, a discordant diagnosis of potential clinical relevance was seen between a small specimen and the follow-up resection. In half of the cases, there was a different small specimen from the same investigational work-up with a concordant diagnosis. Diagnostic inaccuracy was often related to a squamous marker not included in the IHC panel (also seen for the scanned cases), the case being a neuroendocrine tumor, thyroid transcription factor-1 (TTF-1) expression in squamous cell carcinomas (with clone SPT24), or poor differentiation. IHC was used in about 95% of cases, with a higher number of stains in biopsies and in squamous cell carcinomas and especially neuroendocrine tumors. Pre-surgical transthoracic samples were more often diagnostic than bronchoscopic ones (72–85% vs. 9–53% for prevalent types). Conclusions: Although a high overall diagnostic accuracy of small specimens was seen, small changes in routine practice (such as consequent inclusion of p40 and TTF-1 clone 8G7G3/1 in the IHC panel for non-small cell cancer with unclear morphology) may lead to improvement, while reducing the number of IHC stains would be preferable from a time and cost perspective.

## 1. Introduction

In lung cancer, histopathological type influences the selection of predictive testing and treatment. Targetable mutations and fusions are essentially found in adenocarcinomas (AC) and guidelines do not recommend testing of all squamous cell carcinomas (SqCC) [1]. AC and SqCC are both tested for programmed death-ligand 1 (PD-L1) expression, while pemetrexed and bevacizumab are not used as treatment for SqCC [2,3]. Neuroendocrine tumors, consisting of small cell lung carcinoma (SCLC), large cell neuroendocrine carcinoma (LCNEC), and carcinoid tumors are not subject to predictive testing and choice of chemotherapy may differ from non-small cell carcinomas (NSCC) [4]. Hence, accurate and reliable diagnostics is vital, but may be challenging since most lung cancers are not treated surgically due to the advanced stage at diagnosis [5]. While surgical resections provide a solid base for histopathological diagnosis, morphology is less often clear in small biopsies and cytological material, and the addition of immunohistochemical (IHC) markers are often needed to support the diagnosis.

In a previous study, we found a moderate interobserver concordance among 20 pathologists for the diagnosis of non-selective lung and bronchial biopsies based on morphology, thyroid transcription factor-1 (TTF-1), and p40 [6] (the recommended panel for NSCC without distinct features [7,8]). Similar results have been shown in a study on tissue microarrays with a panel of four IHC markers [9].

The main conclusions from our study [6] were that the suboptimal specificity of TTF-1 clone SPT24 may cause diagnostic problems, that neuroendocrine morphology is sometimes missed, and that overuse (as well as occasional underuse) of IHC staining occurs. However, it was not possible in the same study to evaluate real-world diagnostic accuracy when any IHC marker could be ordered.

Although inevitably leading to selection bias, as some lung cancer types are rarely treated surgically, the most reliable method to evaluate diagnostic accuracy in small specimens should include cases with follow-up resections. Such a study would also provide information on usefulness of various sample types. While there is substantial literature on the diagnostic accuracy for detecting a malignant tumor in the lungs for different sampling methods [10,11,12,13], the accuracy for histopathological typing has rarely been assessed [14,15].

The aim of the present study was to evaluate real-world accuracy of lung cancer diagnostics, at two major sites in Sweden, for small specimens with follow-up resection as gold standard, as well as the use of diagnostic IHC staining.

## 2. Material and Methods

### 2.1. Study Population

Consecutive lung resections with a malignant tumor from 1 January 2019 to 31 March 2020 were identified from the pathology departments’ clinical databases in Lund and Stockholm, Sweden. The study population consisted of 268 resections for primary lung carcinomas in Lund and 270 in Stockholm.

During the same period, there were 104 resections in Lund with primary non-epithelial malignancies (*n* = 6), metastatic carcinomas (*n* = 75, including 3 recurrent lung cancers and 44 metastatic colorectal cancers), or metastatic non-epithelial malignancies (*n* = 23). Correspondingly, there were 90 resections in Stockholm with primary non-epithelial malignancies (*n* = 7), metastatic carcinomas (*n* = 59, including 2 recurrent lung cancers and 44 metastatic colorectal cancers), metastatic non-epithelial malignancies (*n* = 23), and one case for which it was unclear whether it was metastasis or primary lung cancer.

For the resected primary lung cancers and corresponding pre-surgical specimens (for both cohorts partly sampled at regional hospitals), data were collected from the pathology departments’ clinical databases, including diagnosis and applied IHC staining. The pre-surgical specimen with the highest number of applied markers was regarded for number of IHC stains if more than one sample had been stained. Predictive IHC markers (e.g., ALK, ROS1, PD-L1) were excluded from all calculations. It was unknown if additional pre-surgical sampling had been performed due to partial clinical work-up outside the region for two cases in the Lund cohort and one case in the Stockholm cohort. To assure diagnostic accuracy of the resected tumors, the slides were reevaluated by the principal investigator (H.B.) for 214 consecutive and 21 additional selected resections.

### 2.2. Scanned Cases

To further investigate the use of IHC markers among consultants working with thoracic pathology, one representative hematoxylin-eosin-stained slide for 11 bronchial and lung biopsies and two representative slides stained with papanicolaou (ThinPrep^®^) and may-grünwald-giemsa for 4 bronchial brushes were scanned on a Hamamatsu NanoZoomer S360 (used for diagnostic scanning at the pathology department in Lund) at the 40× mode.

The cases were selected from the period of investigation to represent typical cases of primary AC with lepidic, acinary, mixed papillary and micropapillary, or mucinous growth pattern, SqCC, NSCC without distinct features (with IHC supporting AC or SqCC), SCLC, LCNEC, metastasis of breast cancer, and metastasis of colorectal cancer.

The participating pathologists were informed that all cases were malignant tumors, as well as the gender, age, and previous malignancy for each case. They were asked to state exactly which diagnostic IHC markers would be ordered. Twelve pathologists in Lund and Stockholm were invited, whereby 10 accepted participation (all co-authors, except H.B., who selected the cases).

### 2.3. Statistics

Number of IHC stains was compared between groups using the Mann–Whitney U test and the Kruskal–Wallis test and analyzed with multiple regression analysis including diagnosis (AC, SqCC, neuroendocrine tumor), sample type (cytology, biopsy), and pathology department (Lund, Stockholm) as variables. A *p*-value < 0.05 was considered statistically significant. The analyses were performed with MedCalc 14.12.0 (MedCalc Software bvba, Ostend, Belgium).

## 3. Results

### 3.1. Cohort Characteristics

Characteristics of the resected primary lung cancers are seen in Table 1. In the Lund cohort, 42 (16%) of the 268 cases had a synchronous primary lung cancer with lower stage in addition to the main tumor, while three (1%) had a synchronous metastasis to the lungs. The stage IV cases both had pleural metastasis, where one case was known but surgery was performed due to persistent pneumothorax while the other proved to be metastasized at surgery. In the Stockholm cohort, 24 (9%) of the 270 cases had a synchronous primary lung cancer with lower stage, while one (0.4%) had a synchronous metastasis to the lungs. One of the stage IV cases presented with a single brain metastasis treated with curative intent while the other proved to be metastasized at surgery.

### 3.2. Diagnostic Accuracy

As evident from Table 1, there was a pre-surgical diagnosis in 164 (61%) of 268 and 174 (64%) of 270 cases in the Lund and Stockholm cohorts, respectively. All cases with a different diagnosis in pre-surgical specimens compared to the resection are summarized in Table 2. Cases with a discrepant diagnosis but both the resection and pre-surgical specimen diagnosed as AC, adenosquamous carcinoma (AdSq), pleomorphic carcinoma with an AC component, or NSCC (not otherwise specified) are presented separately in the table as these diagnoses are essentially handled the same way in the clinical setting. Overall, the most common diagnostic discordance was NSCC on cytology and AC on biopsy and resection with deliberately no IHC staining of the cytological sample or samples (to avoid costly parallel staining), with reference to the biopsy for specified diagnosis pre-surgically. Although a definite diagnosis of AdSq, pleomorphic carcinoma, or combined LCNEC with a NSCC component requires resection, such a diagnosis was (accurately) suggested in four biopsies.

**Figure 1:**
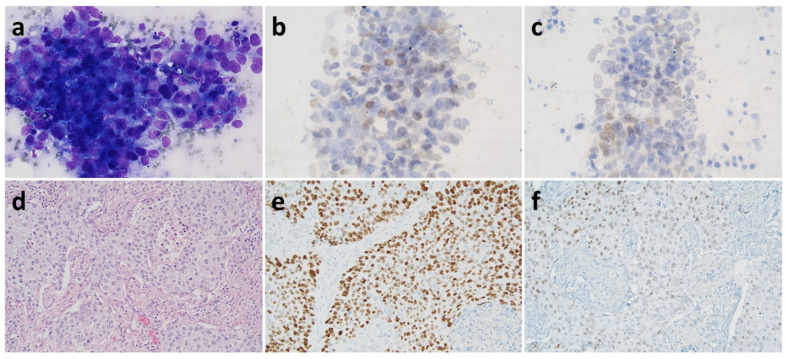
A case diagnosed as adenocarcinoma on pre-surgical cytology (**a**–**c**, ×40 objective) based on TTF-1 positivity with clone SPT24 (no squamous marker included) while the diagnosis proved to be squamous cell carcinoma on resection (**d**–**f**, ×20 objective) with focal positivity with TTF-1 clone SPT24. Staining with (**a**) may-grünwald-giemsa, (**b**,**c**,**f**) TTF-1 clone SPT24, (**d**), hematoxylin-eosin, (**e**) p40.

As evident from Table 2, diagnostic discrepancy of potential clinical relevance was seen in eight (4.9%) of 164 cases in the Lund cohort, but in four of these cases there was a different sample with the same diagnosis as on the resection; in all these cases, there was deliberately no IHC staining of the cytology. The two cases in the Lund cohort with NSCC as diagnosis on biopsy but SqCC on resection were poorly differentiated and negative for cytokeratin 5, TTF-1, and napsin A, while p40 was negative in one and partly positive (20–25%) in the other biopsy. The corresponding number in the Stockholm cohort was eight (4.6%) of 174, with a different sample with correct diagnosis in four of these cases. In all the four cases in the Stockholm cohort where AC was suggested on cytology, but SqCC was seen in the resection, the cytological specimens were stained with TTF-1 clone SPT24, and two were partly (but significantly) positive. In two of the cases other markers were used, but in all four cases no marker for squamous differentiation was included. An example of a case is presented in Figure 1.

### 3.3. Use of Diagnostic IHC Markers

The median number of IHC stains for the 164 and 174 cases with pre-surgical diagnosis in the Lund and Stockholm cohorts were 4 and 2, respectively; the distribution is found in Table 3. The difference was statistically different between the cohorts (Mann–Whitney U test, *p* = 0.003) but not in multiple regression analysis (*p* = 0.54) including sample type (cytology, biopsy) and diagnosis (AC, SqCC, neuroendocrine tumor).

One or more double staining was performed in 91 (55%) and 69 (40%) cases in the Lund and Stockholm cohorts, respectively, and the number of actual slides used for IHC staining (median 3 and 2, respectively) is found in Supplementary Table S1.

In the Lund cohort, there were 42 cases with pre-surgical diagnosis on both biopsy and cytology (as shown in Table 1). In 15 (36%) of these cases, IHC staining was performed on more than one sample. The corresponding number for the Stockholm cohort was 31 (91%) of 34 cases.

The median number of IHC stains for cases with pre-surgical diagnosis on cytological samples only (*n* = 124) compared to cases with diagnosis on biopsy samples only (*n* = 138) from both cohorts were 1 and 4, respectively. The difference was statistically different (Mann–Whitney U test, *p* < 0.0001). The distribution is found in Supplementary Table S2.

The median number of IHC stains for diagnostic pre-surgical specimens where the final diagnosis on resection was AC (*n* = 241), SqCC (*n* = 58), or neuroendocrine tumor (*n* = 30) from both cohorts were 3, 4, and 8, respectively. The difference was significant with Kruskal–Wallis test (*p* < 0.0001) as well as with pairwise Mann–Whitney U test (all *p* < 0.006). The distribution is found in Supplementary Table S3.

Both sample type (cytology, biopsy) and diagnosis (AC, SqCC, neuroendocrine tumor) remained statistically significant factors (both *p* < 0.0001) for number of IHC stains performed in multiple regression analysis also including pathology department (Lund, Stockholm).

The IHC markers suggested by the 10 participating pathologists for the scanned cases are found in Table 4. All pathologists suggested at least one neuroendocrine marker for the three SCLC/LCNEC cases. In the three NSCC cases without distinct morphology, 1–3 pathologists did not include a marker for squamous differentiation (also true for the case with SqCC on cytology), and in one of the cases, one pathologist did not include a marker for adenocarcinomatous differentiation.

### 3.4. Sample Usefulness

Frequency of how often the various pre-surgical sampling procedures were attempted and point at which a malignant diagnosis could be established from the different procedures in the retrospective cohorts is presented in Table 5. As can be seen, transthoracic procedures (fine needle aspirations [FNA] and core biopsies) exhibited a higher diagnostic rate than bronchoscopic procedures among prevalent sampling methods for the surgically treated cases. Moreover, bronchoscopic biopsies had a slightly higher diagnostic rate than bronchoscopic cytology, and, for example, suction catheter or bronchioalveolar lavage were the sole diagnostic sample in only two cases each.

As evident from Table 5, a malignant diagnosis was established in 84 (28%) of 304 bronchial brush samples in the two cohorts. In 25 of these, the diagnosis was NSCC (not otherwise specified), where 18 had a specified diagnosis in a different pre-surgical sample.

Correspondingly, in the 21 (13% of 157) malignant bronchioalveolar lavage samples, the diagnosis was NSCC in six cases, all with a specified diagnosis in a different sample. The numbers for the 23 (17% of 134) malignant suction catheters were 11/9, for the 94 (80% of 118) transthoracic FNAs 4/3, for the 91 (41% of 223) bronchial biopsies 3/3, and for the 122 (79% of 154) transthoracic core biopsies were 1/0.

### 3.5. N2 Metastases

As evident from Table 1, there were 495 cases with one or more surgically sampled N2 stations in the two cohorts. Endobronchial ultrasound (EBUS)-guided lymph node sampling was performed pre-operatively in 193 of these cases and there were five and two cases with metastases to one or two N2 stations, without morphological confirmation of the metastases in pre-surgical specimens. Thus, a metastasis was missed with EBUS in seven (3.6%) cases. In two of these cases, the N2 station with metastasis was not sampled with EBUS (both including station L5), but in the remaining five cases, the EBUS was reported to be representative and without malignancy. Correspondingly, in the 302 cases with no EBUS, there were 11, five, and one case with metastases to a one, two, or three N2 station, respectively. Thus, for the group with no EBUS, N2 metastases were seen in 17 (5.6%) cases (a single metastasis to station L5 in five cases).

## 4. Discussion

Although originating from pathology, this study is also highly relevant for pulmonologists, radiologists, and lung oncologists. Diagnostics of lung tumors involve a team effort relying on sampling (pulmonologists and radiologists) as well as assessment of morphology and ordering of ancillary markers (pathologists). Our study highlights limitations and pitfalls of this diagnostic procedure in daily practice, which should be of interest for oncologists as receivers of pathology reports.

The results support a good overall diagnostic accuracy for lung cancer subtypes for small specimens. However, we can confirm our previous findings that neuroendocrine morphology is sometimes missed, and that TTF-1 clone SPT24 occasionally causes diagnostic problems [6]. Problems relating to TTF-1 clone SPT24 were seen in the Stockholm cohort, while in the Lund cohort, clone 8G7G3/1 was used for histological specimens and clone SPT24 was only used for cytology (due to weaker staining in CytoLyt^®^/PreservCyt^®^-fixed Cellient™-cell blocks). However, based on our results, not including a marker for squamous differentiation and poor differentiation of the tumor may be more important than TTF-1 clone.

Furthermore, slightly more discordant diagnoses were seen for cytology than for biopsies. Inaccuracy with potential clinical relevance was seen in 14 (7%) of 200 cytological specimens and four (2%) of 214 biopsies. However, some cytologies (*n* = 4) were deliberately not stained with IHC as staining was performed on a different specimen. Thus, while these cases were discordant compared to the resection, they cannot be fully defined as inaccurate. Application of both p40 and TTF-1 (preferably clone 8G7G3/1) would most probably have resulted in accurate specified diagnosis in these cases as well as in the cases with a diagnosis of AC on cytology and SqCC on resection. Thus, similar diagnostic accuracy should be possible for cytology and biopsies if there is enough material for IHC staining.

The participating pathologists tended to prefer p40 as a squamous marker, though less seldom staining for CK5 was suggested as well (Table 5). We believe p40 is slightly superior to CK5, in line with the opinion of the WHO group [8]. However, this is only supported by some studies [16,17,18,19], while several reports have shown an essentially equal [20,21,22,23,24,25,26] or inferior [27] sensitivity/specificity profile for p40. A better specificity for TTF-1 clone 8G7G3/1 compared to clone SPT24 has consistently been shown [28,29,30,31,32,33]; we hope the present study contributes to pathology departments in Sweden and elsewhere changing to the clone 8G7G3/1 if a less specific one is being used. Given the limited specificity of neuroendocrine markers [34], routine inclusion of such markers is not recommended in NSCC cases [7]. However, both our present and previous study [6] support a need to improve diagnostics, especially of LCNEC.

While not all pathologists included markers for both squamous and adenocarcinomatous differentiation in cases with NSCC without distinct morphology, the retrospective data as well as the scanned cases at the same time support that unnecessary IHC markers are used in a significant proportion of routine cases. Large panels more often lead to deviant IHC profiles, often without obvious diagnostic gain [23], and result in increased cost and spent time for the involved staff at the pathology department. For example, in the present study CK7 has been used more often than is recommended [7,8], and sometimes additional markers were included based on patient history even if morphology strongly opposes that diagnosis. In Sweden, the recommendation is to stain with TTF-1 also in cases that are obvious non-mucinous AC, which also contributes to a high proportion of cases with IHC staining. However, the balance is difficult as use of IHC clearly leads to more precise diagnoses [35,36,37].

Concerning specimen types, transthoracic samples were more often diagnostic than bronchoscopic samples for our cohorts of surgically treated (i.e., predominantly peripheral) lung cancers, also seen in recent investigations using EBUS-guided bronchoscopy [38,39]. As in our study, a slightly higher sensitivity for transthoracic biopsies than FNA (92% vs. 75%) has been reported [14]. A meta-analysis from 2015 reported a 91% sensitivity for transthoracic needle sampling with similar numbers for CT and ultrasound-guided specimens [40]. We did not have complete information regarding CT or ultrasound guidance and could not compare the two in our study.

Although not the main purpose of the study, our investigation also provided data for the value of EBUS-guided lymph node sampling for detection of N2 metastases, also previously reported [41]. International guidelines recommend combined EBUS and esophageal ultrasound (EUS)-guided lymph node sampling [42]. For our cohorts, we did not have complete information on EBUS vs. EBUS combined with EUS (i.e., EBUS could mean EBUS plus EUS for the cases in our report), thus we could not analyze any difference between the two procedures.

Some additional limitations of the study need to be addressed. Diagnostic accuracy of non-malignant conditions and metastases to the lungs was not evaluated, and this may be of interest in future investigations for an overall accuracy of pulmonary samples. Moreover, we only included cases with follow-up resection (to evaluate diagnostic accuracy), and some of our results may not be applicable to advanced cases. For example, repeated sampling may be performed more often to acquire a specified diagnosis in metastatic disease and bronchoscopic sampling probably has a higher sensitivity in central tumors. Furthermore, no N2 lymph nodes were sampled at surgery in some of our cases (more often in very small lesions or ground glass opacities), although this is unlikely to affect our main results.

In conclusion, our results confirm that TTF-1 clone SPT24 may occasionally cause a diagnostic problem and highlights that both p40 and TTF-1 should always be performed in NSCC without clear morphology. Neuroendocrine and poorly differentiated tumors may also be diagnostically challenging; however, dealing with these problems is less clear, and it is important to be aware of these limitations in pathology. In practice, more IHC are often used than is recommended or needed. Furthermore, in these cohorts of predominantly peripheral tumors, transthoracic procedures were better at confirming a malignant diagnosis. As well, the use of EBUS-guided lymph node sampling leads to more pre-surgically detected N2 metastases.

## Figures and Tables

**Table 1:** Characteristics for consecutive surgically treated primary lung cancer from a 15-month period.

Characteristic	Lund (*n* = 268)	Stockholm (*n* = 270)
Histological type		
Adenocarcinoma	186	213
Squamous cell carcinoma	47	29
Adenosquamous carcinoma	2	5
Large cell carcinoma	1	1
Sarcomatoid carcinoma	3	0
Small cell/large cell neuroendocrine carcinoma incl. combined tumors	10	3
Carcinoid tumor	19	18
Salivary gland type carcinoma	0	1
Stage (TNM8)		
In situ or yT0	7	6
IA	107	138
IB	53	42
IIA	11	13
IIB	58	38
IIIA	23	28
IIIB	7	3
IIIC	0	0
IVA	2	2
IVB	0	0
N Stage		
N0	211	225
N1	43	30
N2	14	15
Number of N2 stations sampled	^a^	
0	30	13
1	25	25
2	42	57
3	134	130
4	33	43
5	4	2
Pre-surgical diagnosis		
Cytology only	24	100
Biopsy only	98	40
Both cytology and biopsy	42	34
No diagnosis	104	96
Frozen section		
Tumor	35	16
Lymph nodes	4	3
Margin/extension	7	1

^a^ For four additional cases in the Lund cohort, N2 lymph nodes were investigated with endobronchial ultrasound (EBUS) but not sampled at surgery.

**Table 2:** Discordant pre-surgical diagnosis for 164 (Lund) and 174 (Stockholm) cases with resection as reference diagnosis.

Discordance	Lund	Stockholm
With potential clinical relevance		
Cytology AC, resection SqCC	0	4 (2)
Cytology NSCC, resection SqCC	4 (4)	0
Cytology AC, resection mixed SCLC/LCNEC	0	1
Cytology NSCC, resection LCNEC	1	0
Cytology LCNEC, resection carcinoid	0	1
Cytology SqCC, resection salivary gland type carcinoma	0	1 (1)
Cytology and biopsy NSCC, resection SqCC	1	0
Cytology and biopsy NSCC, resection mixed SCLC/LCNEC	1	0
Biopsy AdSq, resection SqCC	0	1 (1)
Biopsy NSCC, resection SqCC	1	0
With no/limited clinical relevance		
Cytology NSCC, resection AC	22 (17)	6 (6)
Cytology AC/NSCC, resection AdSq	0	2
Cytology and biopsy NSCC, resection AC	0	1
Cytology and biopsy AC/NSCC, resection pleomorphic carcinoma with AC component	2	0

Number of cases with a different pre-surgical sample with correct diagnosis are represented in parenthesis. AC, adenocarcinoma; AdSq, adenosquamous carcinoma; LCNEC, large cell neuroendocrine carcinoma; NSCC, non-small cell carcinoma (not otherwise specified); SCLC, small cell lung carcinoma; SqCC, squamous cell carcinoma.

**Table 3:** Number of diagnostic immunohistochemical (IHC) markers for the 164 (Lund) and 174 (Stockholm) pre-surgical samples with diagnosis.

IHC Markers	Lund	Stockholm
0	7	8
1	10	55
2	19	29
3	16	6
4	42	15
5	21	8
6	16	3
7	9	9
8	8	3
9	4	2
10	3	11
11–15	6	18
16–20	3	6
21–30	0	1

**Table 4:** The number of participating pathologists suggesting various immunohistochemical markers for the differential diagnosis of 15 cases of pulmonary tumors.

Specimen	Morphology	Previous Malignancy	Age/Gender	10	9	8	7	6	5	4	3	2	1
Core biopsy	AC mucinous	Thyroid papillary micro-cancer	66/F	CK20	CDX2, CK7, TTF-1			Napsin A			PAX8		CA19-9, CK19, HBME1, Ki67, MUC1, MUC2, MUC5AC, MUC6, p40, thyreoglobulin, vimentin
Bronchial biopsy	SqCC	-	67/M				p40	TTF-1	CK5	Napsin A			Ki67, vimentin
Core biopsy	AC acinary	Uterine endometrioid cancer	65/F	TTF-1		Napsin A, PAX8		ER		CK7, PGR		CA125	CK5, CK18, CK19, Ki67, p40, p53, vimentin, WT1
Core biopsy	Metastasis of breast cancer ^1^	Tripple negative breast cancer	49/F	GATA3, TTF-1	Napsin A		p40		CD56, CK5, CK7	Chromogranin A	Synaptophysin	CKAE1/3, GCDFP15, INSM1, mammaglobin, SOX10	CK18, CK19, vimentin
Bronchial biopsy	SCLC	-	69/M	TTF-1	Ki67, Synaptophysin	CD56		CKAE1/3, chromogranin A		Napsin A, p40	CD45, CK5, INSM1		Vimentin
Bronchial biopsy	NSCC ^2^	-	76/F		CK5, p40, TTF-1	Napsin A			CK7				CA19-9, CAIX, CDX2, CK19, CK20, CKAE1/3, GATA3, Ki67, MUC1, MUC2, MUC5AC, MUC6, S100, vimentin
Core biopsy	AC lepidic	Urothelial cancer of the renal pelvis	81/F	TTF-1			Napsin A				CK7, Ki67	GATA3, p40, vimentin	CK5, CK20, CDX2
Bronchial biopsy	Metastasis of colorectal cancer	Rectal cancer	71/M	CK20	CDX2, CK7, TTF-1					Napsin A	SATB2		CA19-9, Ki67, MUC1, MUC2, MUC5AC, MUC6, p40, vimentin
Core biopsy	LCNEC	-	78/F	TTF-1	Napsin A, synaptophysin	CK5, p40	CD56, chromogranin A	Ki67		CK7		INSM1	CK20, ER, GATA3, MUC1, MUC2, MUC5AC, MUC6, PAX8, PGR, vimentin
Bronchial biopsy	NSCC ^3^	-	82/F	TTF-1		Napsin A, p40		CK5, CK7				CDX2, GATA3, Ki67	CA19-9, CAIX, CD56, chromogranin A, CK19, CK20, MUC1, MUC2, MUC5AC, MUC6, PAX8, SOX10, vimentin
Core biopsy	AC micro-papillary/papillary	-	58/F	TTF-1				CK7, napsin A			PAX8	CDX2, GATA3	CA19-9, CK18, CK19, CK20, ER, GATA3, HBME1, Ki67, MUC1, MUC2, MUC5AC, MUC6, p40, vimentin
Bronchial brush	AC	Tounge SqCC	62/M	p40, TTF-1			CK5, napsin A			p16		CK7	CD56, chromogranin A
Bronchial brush	SCLC	-	75/F	TTF-1	Synaptophysin	CD56		CKAE1/3, Ki67, p40	Chromogranin A, napsin A	CK5	CK7, INSM1		CD45
Bronchial brush	NSCC ^2^	-	57/F	TTF-1		Napsin A	p40	CK5	CK7			CD56, synaptophysin	CA125, CA19-9, CDX2, chromogranin A, CK20, p16, ER, GATA3, PAX8, PGR
Bronchial brush	SqCC	Follicular lymphoma	78/F	TTF-1		p40	CK5, napsin A				CK7	CD3, CD20, CDX2	BCL2, BCL6, CA19-9, CD56, chromogranin A, CKAE1/3, Ki67, MUC1, MUC2, MUC5AC, MUC6

^1^ Suggested predictive markers for breast cancer not included in the table; ^2,3^ immunohistochemistry (not available to the participants) favoured AC (^2^) or SqCC (^3^); AC, adenocarcinoma; LCNEC, large cell neuroendocrine carcinoma; NSCC, non-small cell carcinoma; SCLC, small cell lung carcinoma; SqCC, squamous cell carcinoma.

**Table 5:** Pre-surgical sampling with malignant diagnosis/attempted procedures for consecutive surgically treated primary lung cancers.

Sampling Procedure	Lund	Stockholm
EBUS against lymph nodes	16/182 (9%)	1/25 (4%)
Bronchoscopic FNA	2/10 (20%)	3/4 (75%)
Bronchial brush	46/183 (25%)	38/121 (31%)
Bronchioalveolar lavage	3/32 (9%)	18/125 (14%)
Bronchial suction catheter	23/134 (17%)	0/0
Transthoracic FNA	1/2 (50%)	93/116 (80%)
Pleural effusion	1/2 (50%)	0/1 (0%)
FNA against lymph node	0/5 (0%)	0/4 (0%)
Bronchoscopic biopsy	66/176 (38%)	25/47 (53%)
Transthoracic core biopsy	73/86 (85%)	49/68 (72%)
Mediastinoscopy	0/0	0/4 (0%)
Liver biopsy	0/1 (0%)	0/1 (0%)
Brain biopsy	0/0	1/1 (100%)
Head and neck biopsy	0/2 (0%)	0/0

EBUS, endobronchial ultrasound (guided aspirations); FNA, fine needle aspiration.

## Data Availability

Data is available from the corresponding author upon reasonable request.

## Supplementary Materials

The following are available online at https://www.mdpi.com/article/10.3390/biom11111721/s1, Table S1: Number of slides used for diagnostic immunohistochemical staining for the 164 (Lund) and 174 (Stockholm) pre-surgical samples with diagnosis, Table S2: Number of diagnostic immunohistochemical markers for the pre-surgical samples with diagnosis on cytology only (*n* = 124) or biopsy only (*n* = 138), Table S3: Number of diagnostic immunohistochemical markers for the pre-surgical samples with diagnosis of adenocarcinoma (*n* = 241), squamous cell carcinoma (*n* = 58), or neuroendocrine tumor (*n* = 30) on follow-up resection.
